# Influence of fermentation time on proximate composition and microbial loads of Enset, (*Ensete ventricosum*), sampled from two different agroecological districts

**DOI:** 10.1002/fsn3.2527

**Published:** 2021-08-16

**Authors:** Derese Tamiru Desta, Gezhagn Nigusse Kelikay, Meskelu Zekwos, Mesfin Eshete, Hailu Hailemariam Reda, Fikadu Reta Alemayehu, Aemiro Tadesse Zula

**Affiliations:** ^1^ School of Nutrition, Food Science and Technology Academic Center of Excellence for Nutrition Hawassa University Hawassa Ethiopia

**Keywords:** agroecology, enset, fermentation, *kocho*, proximate composition

## Abstract

In southern Ethiopian households, kocho is one of the staple foods which can be kept longer and fermented naturally using locally prepared pits, but evidence about the influences of fermentation of kocho at a different time and agroecology on proximate compositions and microbial loads are limited. Fermented kocho samples at different fermentation times were collected from highland and midland districts of Sidama region of Ethiopia. The standard procedure of AOAC (2005) method was followed. Four microbiological load analyses were conducted. Factorial analysis using JMP 13 was conducted. Across the fermentation time, total carbohydrate, ash, crude protein, and crude fat ranged 36%–40%, 1.9%–3.2%, 3%–4.3%, and 0.1%–0.3%, respectively. The highest total ash content was observed in week one of fermentation both in midland and highland samples. However, in midland, the increment of fermentation time showed a reduction of total ash percentage. Crude protein and fat were observed similar both in midland and highland (*p* > .05). The titrable acidity of Kocho varied from 0.16% to 0.22%. It was shown that it increased in the first three months of fermentation. It was also found to be increased as the fermentation time is increasing. Aerobic mesophilic, lactic acid bacteria, yeast, and mold were highly observed in Kocho as compared to Enterobacteriaceae. The loads varied across the fermentation time. Enterobacteriaceae and yeast and mold count of Kocho decreased with increased fermentation time. In conclusion, agroecology did not affect crude protein percentage as the fermentation time is increased. However, it was shown that fermentation increases protein and fat percentages. The increment of the acidic contents may also suppress the microbial growth for better food safety of kocho products.

## Graphical abstract

In midland, the increment of fermentation time resulted in a reduction of total ash. Crude protein and fat were observed similar both in midland and highland (*p* > .05). Aerobic mesophilic, Lactic acid bacteria, yeast, and mold were highly observed in Kocho as compared to Enterobacteriaceae, and the microbial loads were varied across the fermentation time.

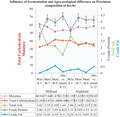

## 1 INTRODUCTION

Enset *(Enseteventricosum*) is categorized under the banana family named as false banana and the most known indigenous crop in Ethiopia. More than 20 million Ethiopian populations depend on Enset for food. Enset is a traditional staple food crop in many parts of the densely populated south and south‐western highlands of Ethiopia(Yemata, 2020). Enset‐based mixed agricultural system is the main agricultural production in Southern Ethiopia(Pijls et al., 1995). Its cultivation represents about 65 percent of the total crop production, and its productivity is very high as compared to other crops in the southern nation, nationalities, and people's regional state of Ethiopia (Birmeta et al., 2004; Mulatu, 2021). It is a multipurpose crop and can be a dependable source of income in areas where it is grown. The major foods obtained from Enset are *kocho* (a bulk of fermented starch obtained from the mixture of decorticated leaf sheaths and grated corm) and bulla (a white powder obtained by squeezing liquid containing starch from the mixture of decorticated leaf sheaths and grated corm). Although many different dishes can be prepared from fermented *kocho*, pancake‐like bread is the most common(Borrell et al., 2020; Yemata, 2020). Enset, a starchy staple crop, is high in carbohydrates, but low in vitamins and protein content, low levels of essential amino acids, such as methionine and isoleucine. In all Enset‐growing areas, Enset is the most frequently served main meal, with a daily average consumption of 0.5 kg, which provides 68% of the total energy intake, 28% iron, but no vitamin A content(Borrell et al., 2020). The nutritional composition depends on factors such as type or variety of the Enset (Bosha et al., 2016; Gizachew et al., 2019; Yemata, 2020). *Kocho* and bulla are the two major foods produced from Enset in Ethiopia. Enset serves as a staple diet; hence, Enset foods have both nutritional and cultural values for the society (Olango et al., 2014). Enset is prepared either as *kocho* (fermented and bread‐like food, a fermented product of the corm and pseudostem), *bulla* (dehydrated juice collected during decortication of the pseudostem and grating of the corm, thereafter rehydrated from concentrate and prepared as pancake or porridge), or *amicho*(boiled corm pieces, eaten like a potato). Various Enset‐based recipes include the addition of spices, milk, maize flour, butter, beans, or cabbage (Olango et al., 2014).

Limited evidence shows that fermentation time has a significant influence on the average carbohydrate content. The moisture content decreases as the fermentation time increases, and it is known that the concentration of moisture has a direct relation to the shelf life of the end product as a conducive environment is created for microbial growth (Ashenafi, 2008; Urga et al., 1997; Zewdie et al., 2011). The physicochemical properties of *kocho* may vary due to different factors. The processing practices including fermentation periods and agroecological conditions attribute to the variations(Yemata, 2020).

In the Sidama and Southern Ethiopian households, *kocho* is the staple food and is kept longer and fermented naturally using locally prepared pits. However, there is limited evidence about the effect of different fermentation times on proximate compositions and microbial loads. Therefore, the objective of the current study is to evaluate the influence of the fermentation period on the proximate composition and selected microbial loads of *kocho*.

## 2 METHODS AND MATERIALS

### 2.1 Study setting and sample collections

The *kocho* samples for analysis were collected from two different agroecological settings of Sidama regional state. Based on the agroecological classifications and availability(Eshete et al., 2020) of *kocho* products, Hula district, as highland (>2,300 m a.s.l), Bensa and Dale districts as midlands (1750–2300 m a.s.l) were purposively selected. Fermented *kocho* samples having different fermentation times were collected. The six subdistricts (smallest administrative unit in Ethiopia) were selected from the three randomly selected districts of Sidama regional state. Representative samples from the households representing different fermentation times were collected from each of the subdistricts. The edible part (flesh and skin) was transferred into plastic bags and kept in an icebox. The collected samples from the two agroecologies were transported to the Hawassa University School of Nutrition, Food Science and Technology laboratory and analyzed for proximate and microbial composition. The laboratory analyses were conducted for each of the agroecological conditions.

### 2.2 Proximate analysis

The proximate composition (moisture content, crude protein, crude fat, and total ash) of collected *kocho* samples was determined following the procedure of AOAC (2005) methods(Al‐mentafji, 2006).

### 2.3 Microbial Load

The microbiological load analysis of *Kocho* for Enterobacteriaceae, aerobic mesophilic count, lactic acid bacteria, and yeast and mold parameters were done following a standard procedure. About 10g of *Kocho* sample was taken and homogenized in 90 ml sterilized 0.1% peptone water, and the sample was diluted in series of ten‐fold dilutions. Diluted samples from appropriate dilutions were spread in duplicate on predried agar plates of appropriate media for counting. Aerobic mesophilic count (AMC) was enumerated on plate count agar (PC) after incubation at 30^0^c for 48hours. Enterobacteriaceae (EB) were enumerated on Macconkey agar and counted the colony after 24hour incubations. Lactic acid bacteria were enumerated on de‐Mann, Ragosa, Sharpe (MRS) agar plates after incubation at 30^0^c for 48hours. Yeast and mold were enumerated in yeast extract glucose chloramphenicol bromophenol blue agar (YGCA) at 28^0^c for 5 days. The result was calculated as the following formula. The microbial load was reported in cfu/g.

$$\text{Cfu/g}=\frac{average\;number\;of\;colony\;per\;duplicated\;plate}{dilution\;factor\;\times \;volume\;factor}$$

### 2.4 Data Management and Analysis

After the laboratory analysis, all data were compiled into a spreadsheet and analyzed using JMP Pro 13 software. Means and standard deviations for the continuous data were determined. Analysis of variance/factorial analysis was conducted to see the effect of fermentation time and agroecological differences. The mean separation was done using Tukey's HSD test at *p* <.05.

## 3 RESULTS AND DISCUSSIONS

### 3.1 Proximate composition of *Kocho* fermented at different fermentation time

The proximate composition of *kocho* influenced by the fermentation time is indicated in Table 1. The moisture content of *Kocho* varied from 53.79% in T3 to 57.63% in T2. Semman (2019) and his colleagues reported a similar range across different clones of *Kocho* and bulla products. The current study showed the highest percentage of moisture content was observed in T1 (57.07%) and T4 (56.19%) of fermentation times. However, the moisture content decreased as the fermentation time increases. The decrement of moisture content toward the increase of fermentation time might be due to the dry matter content had a chance to increase during fermentation as a result of microbial proliferation (Omojasola, 2000). Other previous studies have shown that the moisture content decreases as the fermentation time increases (Urga et al., 1997; Geel, 2018; Andeta et al., 2018).

**TABLE 1:** Effects of fermentation time on proximate composition of kocho sampled from Sidama Regional State, Ethiopia (July 2020)

Fermentation time	Moisture (%)	Titrable Acidity	Total Ash (%)	Crude Protein (%)	Crude Fat (%)	Total Carbohydrate (%)
T1	57.07 ± 4.97^a^	0.18 ± 0.07^c^	3.24 ± 0.44^a^	3.085 ± 0.31^b^	0.135 ± 0.14^d^	36.47 ± 5.04^ab^
T2	57.6375 ± 0.58^a^	0.2275 ± 0.03^a^	2.1725 ± 0.08^b^	4.2975 ± 0.56^a^	0.295 ± 0.04^c^	35.5975 ± 0.27^b^
T3	53.7975 ± 2.45^b^	0.165 ± 0.01^d^	2.21 ± 0.12^b^	4.05 ± 0.23^a^	0.32 ± 0.31^a^	39.6225 ± 2.40^a^
T4	56.1925 ± 3.08^ab^	0.2 ± 0.03^b^	1.86 ± 0.49^c^	4.2875 ± 0.29^a^	0.315 ± 0.24^b^	37.345 ± 3.63^ab^

Note

Where T1 is *Kocho* fermented for 1 week, T2 is *Kocho* fermented for 3 months, T3 is *Kocho* fermented for 6 months and T4 is *Kocho* fermented for more than a year. The levels are mean±standard deviations. Mean followed by different superscript within the column are significantly different at *p* <.05.

Titratable acidity (TA) is the amount of acid present in a certain sample determined with titration against an alkali. The titrable acidity of *Kocho* varied from 0.16% to 0.22%. The study showed that the titrable acidity of *Kocho* was increased in the first three months of fermentation, and similarly, the titrable acidity also increased as the fermentation time is increasing. The higher titrable acidity toward the increase of fermentation time could be attributed to the accumulation of some organic acid and acetic acid resulting from the activities of some fermentative organisms such as lactic acid in the fermenting foods (Obadina et al., 2013).

The total ash percentage on the other hand was decreased from 3.24% to 1.86% as the fermentation time increases from T1 to T4. The study showed that T2 and T3 had similar (*p* >.05) ash content with each other, but T2 and T3 had lower (*p* <.05) ash content as compared to T1 and higher (*p* <.05) ash content as compared to T4. The lower ash content toward the increase of fermentation time could be attributed to leaching of the minerals through permeable fermentation pit, and this is observed by different scholars. However, similar percentage of total ash content across different varieties and fermentation time were indicated (Bekele, 2015; Gizachew et al., 2019). Similar total ash content was reported by other studies (Fanta & Neela, 2019). Higher percentages of ash content was observed in this study as compared to the study reported by(Bekele, 2015; Semman et al., 2019; Urga et al., 1997).

The crude protein of *Kocho* was increased significantly (*p* <.05) from 3.08% to 4.28% as the fermentation time was increased from T1 to T4. Our study showed that T2, T3, and T4 had similar crude proteins to each other. However, the samples had significantly higher (*p* <.05) crude protein content as compared with *Kocho* fermented for a week. Hence, the crude protein percentage increased as a result of the fermentation process, and this might be due to experienced women adds different food groups to enhance the fermentation processes. The other reason for the increase of crude protein toward the increase of fermentation time could be due to some fermentation results from anabolic processes leading to polymer build‐up or due to microbial cell proliferation(Obadina et al., 2013), and fermentation may result in an apparent increase in nonprotein nitrogen and free amino acids which was indicated (Odibo et al., 1990; Urga et al., 1997). However, contrary to this study, other studies have observed that the fermentation process decreases the protein contents linked to the leaching of more soluble proteins and amino acids (Besrat et al., 1979; Urga et al., 1997).

In the current study, the crude fat content of *Kocho* was found very low, 0.5%. The study showed that the crude fat content was significantly increasing at (*p* <.05) from 0.13% to 0.3% as the fermentation time is increasing from T1 to T4. However, contradicting finding was reported from research conducted by others (Astuti et al., 2000). Other study reported that the longer fermentation of the product could produce short chain fatty acid (hexanoic acid) due the presence of *Caproiciproducens* species(Weldemichael et al., 2019). This could probably result an increase of crude fat.

**FIGURE 1:**
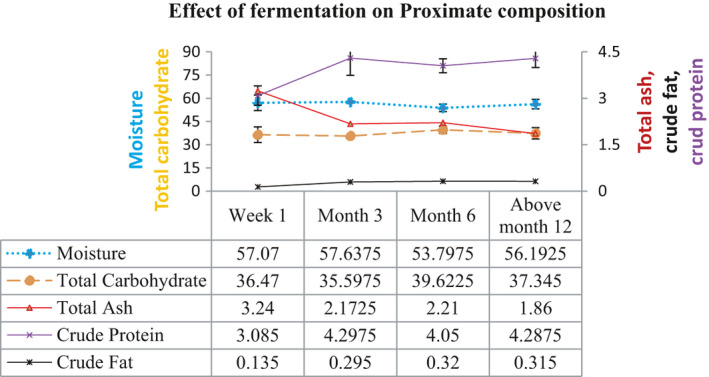
Effect of fermentation time on proximate composition of *Kocho*

The carbohydrate content of the *Kocho* in the current study is considerably varied from 35.59% to 39.62%. The study showed that only T2 and T3 are significantly different (*p* <.05), and the higher carbohydrate content was observed in T3, whereas T2 had lower carbohydrate content. The variation in carbohydrate content might be due to the use of different methods of analysis to determine the total carbohydrate content (Figure 1).

### 3.2 Fermentation time effect on proximate composition of *Kocho* from two Agroecological districts

The study revealed fermentation period affected the proximate composition of the Kocho. The effect of fermentation time on the proximate composition from two agroecological districts is indicated in Table 2. The moisture content of *Kocho* fermented with a different fermentation time from the two agroecological districts is varied considerably from 51.67% to 60.94%. The study showed 1st week fermented *Kocho*from the midland agroecology had higher (*p* <.05) moisture content, and, however, it was not significantly different with *Kocho* fermented for 3 months from the midland agroecology and *Kocho* fermented for 3 months, 6 months, and more than a year from the highland agroecology. In contrary to this, our study indicated that *Kocho* fermented for 6 months from the midland agroecology had a lower (*p* <.05) moisture content, even though it was not significantly different with *Kocho* fermented for more than a year from the midland agroecology, and *Kocho* fermented for a week and 6 months from the highland agroecology. In general, the moisture content of *Kocho* was decreased in the midland agroecology as the fermentation time is increases; however, the reverse was indicated in the highland agroecology, and the moisture content was increased as the fermentation time was increased. Even if there is limited evidence about the effect of agroecology on moisture content of *kocho*, the result was assumed to be influenced by the agroecological systems. Other study conducted on Ethiopian pea (*Pisum sativum var. abyssinicum A. Braun*) has shown that the variation of moisture contents of soil across altitudinal changes. The highland areas are cooler than midlands resulting in higher moisture resulting in an increment of moisture content(Gebreegziabher & Tsegay, 2020).

**TABLE 2:** Effects of agroecological differences and fermentation time on proximate composition of *kocho* sampled from Sidama Regional State, Ethiopia (July 2020)

Agroecology	Fermentation time	Moisture (%)	Total Ash (%)	Crude Protein (%)	Crude Fat (%)	Total Carbohydrate (%)
Midland	T1	60.945 ± 4.06^a^	3.62 ± 0.85^a^	2.86 ± 0.75^a^	0.01 ± 0.23^g^	32.565 ± 4.16^c^
	T2	57.64 ± 0.54^abc^	2.18 ± 0.14^c^	4.25 ± 0.85^a^	0.26 ± 0.00^d^	35.67 ± 0.17^bc^
	T3	51.675 ± 0.19^d^	2.105 ± 0.01^c^	3.95 ± 0.28^a^	0.59 ± 0.00^a^	41.68 ± 0.47^a^
	T4	53.535 ± 0.06^bcd^	1.44 ± 0.04^d^	4.425 ± 0.32^a^	0.11 ± 0.00^e^	40.49 ± 0.21^ab^
Highland	T5	53.195 ± 1.56 cd	2.86 ± 0.03^b^	3.31 ± 0.04^a^	0.26 ± 0.00^d^	40.375 ± 1.55^ab^
	T6	57.635 ± 0.86^abc^	2.165 ± 0.01^c^	4.345 ± 0.45^a^	0.33 ± 0.00^c^	35.525 ± 0.4^bc^
	T7	55.92 ± 0.1^abcd^	2.315 ± 0.01^c^	4.15 ± 0.18^a^	0.05 ± 0.00^f^	37.565 ± 0.28^abc^
	T8	58.85 ± 0.42^ab^	2.28 ± 0.07^c^	4.15 ± 0.28^a^	0.52 ± 0.00^b^	34.2 ± 0.07^c^

Note

Where T1 is *Kocho* fermented for 1 week, T2 is *Kocho* fermented for 3 months, T3 is *Kocho* fermented for 6 months, and T4 is *Kocho* fermented for more than a year in Midland agroecology. T5 is *Kocho* fermented for 1 week, T6 is *Kocho* fermented for 3 months, T7 is *Kocho* fermented for 6 months, and T8 is *Kocho* fermented for more than a year in Highland agroecology. The levels are mean±standard deviations. Mean followed by different superscript within the column are significantly different at *p* <.05.

The interaction of agroecological difference and fermentation period affects the total ash content, and the total ash content is varied considerably from 1.44% to 3.62%. The study showed *Kocho* fermented for one week from the midland agroecology had higher total ash content as compared with *Kocho* fermented for one week from the highland agroecology. Similarly, *Kocho* fermented for one week from the midland and highland agroecology had a higher (*p* <.05) total ash content as compared with the total ash content of the rest *Kocho*. The higher total ash content of Kocho fermented for one week in both agroecology could be due to high amount of fiber is expected and the product is not degraded yet, since fermentation is at the start point. In conclusion of the total ash content of fermented *Kocho* from the two agroecology differences indicted that the total ash content was decreased toward the increase of fermentation time.

The crude protein content of *Kocho* influenced by the fermentation period and the agroecological difference is varied from 2.86% to 4.42%. However, the study showed that the crude protein content of *Kocho* was not significantly affected by the interaction of fermentation time and agroecological difference.

The fat content of *Kocho* from the interaction of fermentation period and the agroecological difference is varied significantly from 0.01% to 0.59% at *p* <.05. The study revealed that *Kocho* fermented for six months from the midland agroecology and *Kocho* fermented for more than a year from the highland agroecology are significantly different from each other (*p* <.05), but they had also a higher (*p* <.05) crude fat content as compared with rest *Kocho*. In contrary to this, *Kocho* fermented for more than a year from the midland agroecology and *Kocho* fermented for six months from the highland agroecology had lower (*p* <.05) crude fat content. In general, the higher fermentation period in both agroecology had higher crude fat content and this could be due to fermentation break the nutrient composition.

**FIGURE 2:**
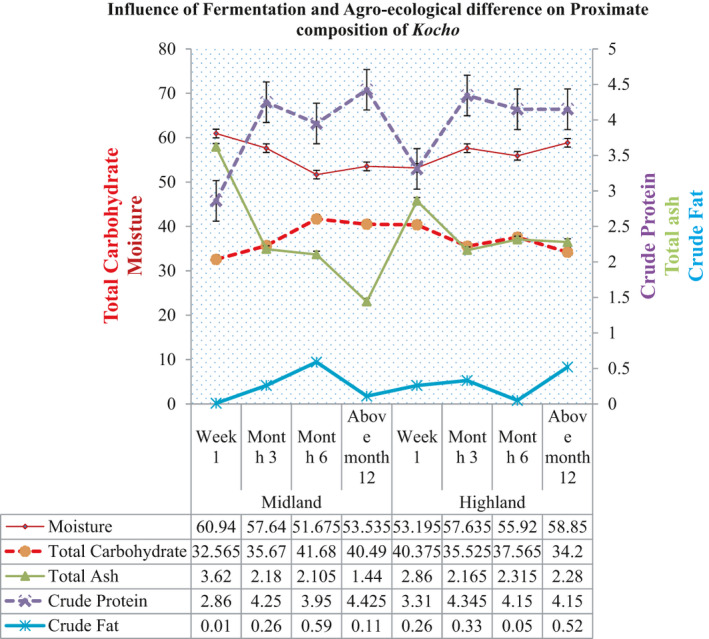
Effect of fermentation time on proximate comparison of *kocho* the midland and highland agroecology

The total carbohydrate content of *Kocho* was varied from 32.56% to 41.68%, and the interaction showed a significant effect on the total carbohydrate content of *Kocho*. The study revealed that *Kocho* fermented for six months from the midland agroecology had higher total carbohydrate, even though it was not significantly different with *Kocho* fermented for more than a year from the midland agroecology and *Kocho* fermented for a week and 6 months from the highland agroecology. Conversely, *Kocho* fermented for a week from the midland agroecology and *Kocho* fermented for more than a year from the highland agroecology had lower total carbohydrate content. Our study revealed that the total carbohydrate content of *Kocho* from the midland agroecology was increased as the fermentation time was increased. However, in the highland agroecology, the total carbohydrate content was decreased as the fermentation time increased. The total carbohydrate content was determined using the difference method, and this could be a factor for the variation of total carbohydrate content (Figure 2). The variation in proximate composition of *kocho* from different agroecology might due to the phenotypic plasticity of enset. According to Almaz Negash et al. (2002), enset is known for its ability to adapt to different agroecological conditions, which can influence its nutritional composition.

### 3.3 Microbial counts of *kocho* sample at different fermentation time

**FIGURE 3:**
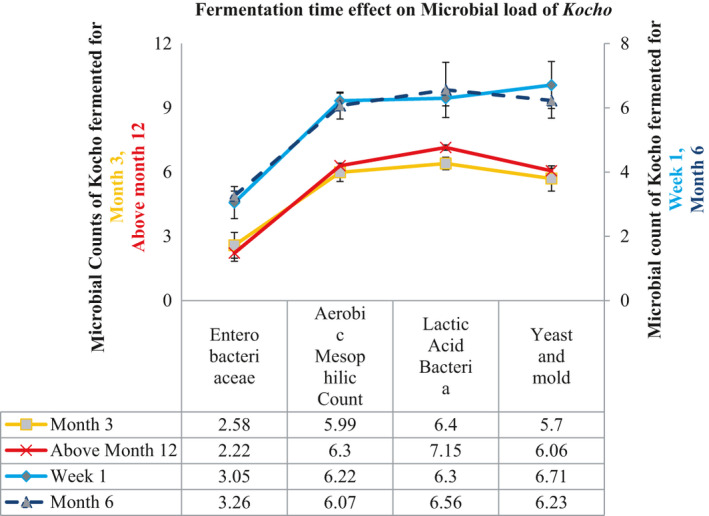
Selected microbial load across fermentation time of *kocho* sample

The microbial load of *Kocho* was indicated in (Figure 3). The key factors which may influence microbial growth were observed in the current study, and the factors were crude protein, titrable acidity, and moisture content. Our study showed that aerobic mesophilic, lactic acid bacteria, yeast, and mold were highly observed in *Kocho* as compared to Enterobacteriaceae, and the microbial loads were affected by the fermentation time. Above all, lactic acid bacteria and yeast and mold growth were highly observed and affected by the fermentation time. The mean of the aerobic mesophilic count was comparable at week one (6.2 log cfu/g) and month twelve (6.3 log cfu/g), and at month three and month six, the lowest aerobic mesophilic count was observed. The study revealed aerobic mesophilic count was not significantly varied toward the increase of fermentation time.

Regarding yeast and mold count, the study showed *Kocho* fermented for more than three months had low yeast and mold count; however, other studies support the growth of yeast and mold in fermented Enset, but at some points, there might be spikes and decreases (Andeta et al., 2018; Karssa et al., 2014). Similarly, *Kocho* fermented for more than a year had a low Enterobacteriaceae count, and the mean for Enterobacteriaceae was varied from 2.22 log cfu/g to 3.26 log cfu/g. The minimum count for Enterobacteriaceae was observed in *Kocho* fermented for more than a year. The lowering of Enterobacteriaceae and yeast and mold count of *Kocho* toward the increase of fermentation time could due to the increase of titrable acidity which is indicated in the study.

Several studies prove that the Enterobacteriaceae count decreases as the fermentation time increases, even it is not detectable at some points (Andeta et al., 2018; Birmeta et al., 2019; Karssa et al., 2014). However, probably linked to hygienic practices either during the sampling or unhygienic condition of the pit in the current study, it was more than detectable counts throughout the fermentation time. The mean lactic acid bacteria count was varied from 6.3 log cfu/g to 7.15log cfu/g, and the study showed that the lactic acid bacteria count was increased as fermentation time is increased, and this could also be associated with the increase of titrable acidity when the fermentation time is increased since titrable acidity create favorable condition for lactic acid bacteria and this is supported by another study (Andeta et al., 2018).

## 4 CONCLUSIONS

In our current study, it can be concluded that in the first week of fermentation time, the *kocho* sample acquired the highest total ash percentage both in midland and highland samples. The ash percentage was decreased as a fermentation time increases in midland but not in the highland *kocho* sample. It is indicated that crude protein was not affected by the agroecology at different fermentation times. Aerobic mesophilic, lactic acid bacteria, yeast, and mold were highly observed in *Kocho* as compared to Enterobacteriaceae, and the microbial loads were affected by the fermentation time. This study proves that fermentation improves protein and fat percentages. The increment of the acidic contents may also suppress the microbial growth for better food safety of *kocho* products.

## ETHICAL APPROVAL

The current research does not include humans. But while *Kocho* sample collection consent was taken from the fermenters.

## Data Availability

The datasets used and/or analyzed during the current study are available from the corresponding author on reasonable request.
